# Segregating memories: targeting microenvironment of neuronal ensembles

**DOI:** 10.1038/s41392-022-01214-5

**Published:** 2022-10-12

**Authors:** Ying Liu, Miao Xu, Wei-Guang Li

**Affiliations:** Department of Rehabilitation Medicine, Huashan Hospital, Institute for Translational Brain Research, State Key Laboratory of Medical Neurobiology and Ministry of Education Frontiers Center for Brain Science, Fudan University, 200032 Shanghai, China

**Keywords:** Cellular neuroscience, Neurological disorders

A recent study by Shen et al. reported that a delayed augmentation in the neuronal expression of C–C chemokine receptor type 5 (CCR5)—a well-known immune molecule in the peripheral inflammatory microenvironment—after the acquisition of a contextual memory determines the duration of the temporal window for linking with another memory, effectively elucidating mechanisms that shape the memory linking timescales.^1^

Memories are acquired in a particular context, often relate to a previous experience, and affect subsequent memory formation. Memory linking is the process that integrates related memories into an associative mnemonic structure of interconnected representations. Typically, memories sharing certain attributes are dynamically interlocked, such that one memory increases the likelihood of retrieval of another. Three main factors facilitate the memory linking: time, space, and perceptual/conceptual similarities.^2^ Mechanistically, overlapping neuronal ensembles for different memories—a set of learning-activated cells encoding the initial memory and biased to be allocated toward a subsequent memory^3^—may be crucial in the formation of mnemonic structures.^2^ Temporally distinct memory links appear to use biological substrates that govern varied timescales to dictate how different memories collaborate to create fluid memory representations, although the biological substrates themselves remain not fully identified.

Shen et al. utilized contextual fear conditioning (Fig. 1a) as a behavioral paradigm to assess memory linking.^1,3^ Mice were first exposed to a distinct context (context A), then were placed in another context (context B) after variable timeframes. Two days later, mice were again exposed to context B where they received electric footshocks. Two days after footshock, mice were finally returned to context A for a test of freezing behavior—a behavioral fear response and an indicator of memory linking between contexts A and B, since the mice received footshock only in context B. The mice with a 5-h temporal window between exposure to contexts A and B showed a significant fear response in context A, indicating effective memory linking (Fig. 1b). In contrast, mice with longer temporal windows (24 h or longer) failed to develop memory linking (Fig. 1c). Thus, memory for one context can be linked to memory for a subsequent context when temporally related.

**Fig. 1:**
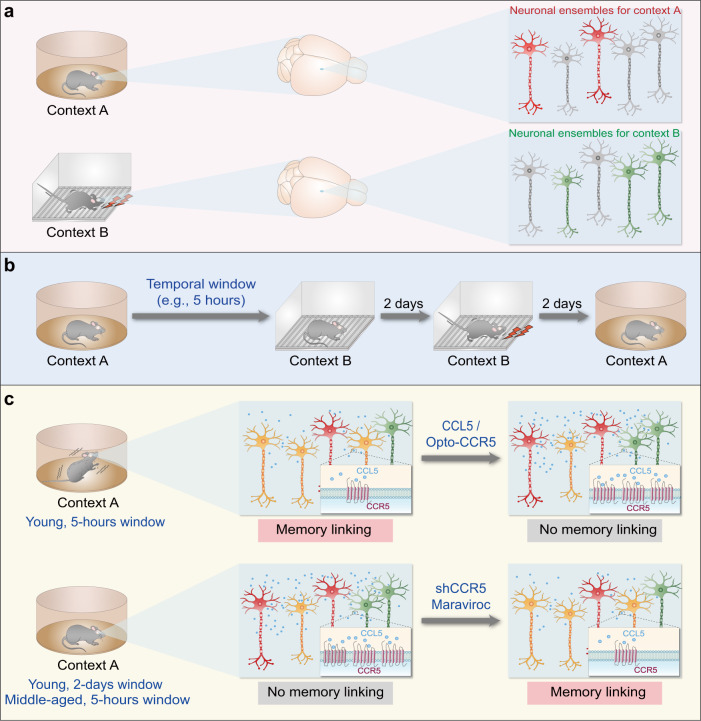
CCR5 regulates the overlap of neuronal ensembles for different memories, thereby altering the temporal window for memory linking. **a** Distinct neuronal ensembles are responsible for memories in different contexts. The red- and green-colored neurons represent neuronal ensembles for memories of context A and context B, respectively. **b** A modified contextual fear conditioning as the behavioral paradigm to assess memory linking. Mice were placed in context A, followed by a changeable temporal window (5-h as a typical temporal window) before being placed in context B. Two days later, the mice received footshocks in context B. The presence of a significant fear response in context A indicates effective memory linking. **c** The activity and expression levels of CCR5 determine the temporal window for memory linking. While young adult mice with a 5-h temporal window between exposure to contexts A and B showed effective memory linking, mice with the same temporal window but with ectopic activation of CCR5 in the hippocampal CA1 region or injected with CCL5 protein showed insignificant memory linking. Likewise, young adult mice with a longer temporal window (up to 2 days) showed insignificant memory linking. Middle-aged mice displayed higher CCR5 expression in the hippocampal CA1 region and failed to show memory linking in the 5-h group, which was reversed following administration of the FDA-approved CCR5 antagonist maraviroc. The orange-colored neurons represent the overlapped neuronal ensembles that link memories of context A and context B

To determine mechanisms that close the temporal window for memory linking to segregate temporally distinct events, Shen et al. considered the potential role of CCR5. In peripheral tissues, CCR5 is well known as an immune receptor protein in the inflammatory microenvironment and as a co-receptor for HIV infection.^1^ CCR5 is enriched in the hippocampal CA1 region and serves as a negative regulator of cyclic-AMP-response-element-binding protein (CREB) activation as well as neuronal excitability.^4^ In the current study, Shen et al. observed that mRNA levels of CCR5 and its ligand CCL5 increased until 12 h after contextual fear conditioning and fell back to baseline levels in hippocampal CA1 region (which is critical for both learning and memory linking) within 24 h, suggesting that CCR5 may adjust the temporal window of memory linking.

Shen et al. then bidirectionally manipulated the activity of CCR5 signaling to examine the effects on the temporal window for contextual memory linking. Both optical activation of a photosensitive form of CCR5 and injection of CCL5 protein compromised memory linking under specific temporal windows that would otherwise allow successful memory linking (Fig. 1c). Conversely, mice with genetic knockdown of CCR5 in hippocampal CA1 region showed a much longer timescale for memory linking (Fig. 1c). Thus, the activity of CCR5 signaling is negatively correlated with the timescales of memory linking.

By investigating the potential mechanisms of CCR5 in memory linking, Shen et al. found that optically activating CCR5 significantly decreased learning-induced expression of c-Fos, a characteristic marker for neuronal ensembles of memory, in the hippocampal CA1 region. CCR5 also steadily decreased the overlap between neuronal ensembles activated in both the original and linked memories of two contexts, which was consistent with the real-time calcium activity of hippocampal CA1 neurons in vivo. Collectively, CCR5 signaling decreases neuronal excitability, which prevents subsequent memories from being distributed to the initial memory ensembles, thereby reducing the overlap between the two memory ensembles and consequently diminishing memory linking. Notably, CCR5 appears to serve as a suppressor for neuronal excitability to trigger one memory retrieval via another memory and therefore closes the temporal window for memory linking.

In addition, Shen et al. observed that the hippocampal *Ccr5* and *Ccl5* mRNA expression levels were higher in middle-aged mice compared to their younger counterparts in the home cage. Furthermore, the middle-aged mice had a shorter temporal window than young mice, suggesting a progressing deficit in memory linking during aging. Both genetic deletion of CCR5 and pharmacological blockade of CCR5 activity with maraviroc (an FDA-approved CCR5 antagonist for HIV treatment)^1^ ameliorated memory linking deficits in middle-aged mice, indicating that targeting CCR5 significantly improves contextual memory linking during aging.

In summary, this exciting work by Shen et al. revealed an unprecedented role of CCR5 in closing the temporal window for memory linking (Fig. 1c). This discovery has significantly broadened the scientific horizon of memory linking research. First, properly extending or shortening the temporal window for memory linking may contribute to remedies for amnesia in Alzheimer’s disease or traumatic flashbacks in post-traumatic stress disorder. Second, CREB-dependent upregulation of neuronal excitability governs memory allocation, which facilitates memory linking to form an associative mnemonic structure. The collaboration between CREB-dependent excitability control and local synaptic mechanisms is expected to be further investigated.^5^ Third, given that CCR5 is present in the microenvironment of memory ensembles, just as it is present in the microenvironment of peripheral inflammation, the potential roles of the broadly distributed extracellular matrix are worth considering. Targeting the neuroglia and perivascular cells within the microenvironment and their connectivity signaling cascades with neuronal ensembles may inform strategies in clinical therapies that could alter memory ensembles in mnemonic structures, thus affecting the storage, linking, and retrieval of two or more memories.

## References

[1] Shen Y (2022). CCR5 closes the temporal window for memory linking. Nature.

[2] de Sousa AF, Chowdhury A, Silva AJ (2021). Dimensions and mechanisms of memory organization. Neuron.

[3] Cai DJ (2016). A shared neural ensemble links distinct contextual memories encoded close in time. Nature.

[4] Zhou M (2016). CCR5 is a suppressor for cortical plasticity and hippocampal learning and memory. eLife.

[5] Lisman J, Cooper K, Sehgal M, Silva AJ (2018). Memory formation depends on both synapse-specific modifications of synaptic strength and cell-specific increases in excitability. Nat. Neurosci..

